# Experimental Study on the Mechanical Properties and Microstructure of Metakaolin-Based Geopolymer Modified Clay

**DOI:** 10.3390/molecules27154805

**Published:** 2022-07-27

**Authors:** Xianzeng Shi, Qingkun Zha, Shuqing Li, Guojun Cai, Dun Wu, Chaojiao Zhai

**Affiliations:** 1 Key Laboratory of Intelligent Underground Detection Technology, Anhui Province Key Laboratory of Advanced Building Materials, College of Civil Engineering, Anhui Jianzhu University, Hefei 230601, China; shixianzeng@ahjzu.edu.cn (X.S.); zqk17685841763@163.com (Q.Z.); zcj@ahjzu.edu.cn (C.Z.); 2 Sinosteel Maanshan General Institute of Mining Research Co., Ltd., Maanshan 243000, China; lishuqin03@foxmail.com

**Keywords:** clay, metakaolin, geopolymer, sodium hydroxide (NaOH), unconfined compressive strength, scanning electron microscopy (SEM), X-ray diffraction (XRD)

## Abstract

Clay is found in some countries all over the world. It usually has low compressive strength and cannot be used as a bearing material for subgrade soil. In this paper, the influence of basicity on a metakaolin-based polymer binder to improve clay was studied. The effects of the molar concentration of the alkali activator, different concentration of the metakaolin-based geopolymer and curing time on unconfined compressive strength were studied. The alkali activator-to-ash ratio was maintained at 0.7. The percentage of metakaolin added to the soil relative to metakaolin and soil mixture was 6%, 8%, 10% and 12%. The sodium hydroxide concentrations are 2M, 4M, 6M and 8M. Unconfined compressive strength (UCS) was tested on days 3, 7, 14 and 28, respectively. Compared with original clay, the results show that the unconfined compressive strength increases with the increase in metakaolin content and molar concentration of NaOH. The maximum compressive strength of the sample with NaOH concentration of 8M and percentage of 12% was 4109 kN on the 28th day, which is about 112% higher than that of the original clay. Scanning electron microscopy (SEM) and X-ray diffraction (XRD) results showed that the cementing compound covered the clay particles due to the reaction of the geopolymer with the clay, resulting in the formation of adhesive particles. The main purpose of this study is to verify the effectiveness and stability of metakaolin-based geopolymer binder polymerization under normal temperature and a strong alkali environment. The results can provide parameters for the application and promotion of metakaolin-based geopolymers in soil improvement engineering.

## 1. Introduction

Clay is widely distributed in the world and is inevitably encountered in the process of road and railway construction in China. Due to the weak microstructure bonding of clay particles, the superstructure is vulnerable to major damage (e.g., cracking, subsidence, collapse) [1]. Clay is widely distributed in the world. In the process of road and railway construction in China, it is inevitable that clay will increase in volume when it becomes wet and shrinks, becoming difficult to compress due to water loss, making it unsuitable as a subgrade. Soil improvement includes physical improvement, chemical improvement and non-traditional active agent improvement. In chemical methods, in order to improve strength, many scholars have adopted various cementing materials for stabilization, including Portland cement, lime, steel slag and fly ash. Chemical additives are one of the suitable methods to improve clay soil. Conventional soil stabilizers (i.e., cement, lime, etc.) are widely used around the world. The main disadvantage of conventional stabilizers is that their production processes are energy intensive and emit large amounts of carbon dioxide (CO_2_), which exacerbates global environmental pressures [2].

China has rich kaolin mineral reserves. Kaolin mineral resources rank among the top in the world, with 267 proven mineral sites and 2.91 billion tons of proven reserves, which are widely used in the light industry, chemical industry and building materials. Road engineering needs an economical and environmentally friendly way to reinforce the soil. When the subgrade soil is found to be clay, this brings about construction challenges, which is one of the problems faced by many geotechnical engineers.

Suraj D. Khadka et al. [3] synthesized two forms of stabilizer, one using metakaolin (MK) and the other using fly ash (FA), as aluminosilicate precursors to stabilize high plastic clays. Abdila S R et al. [4] investigated soil stabilization using GGBS and fly ash-based geopolymer processes. April Anne S. Tigue et al. [5] used geopolymer precursors such as fly ash and volcanic ash to stabilize the soil. Hsiao Yun Leong et al. [6] added fly ash to residual soil to produce soil–fly ash geopolymer bricks. Mazhar Syed et al. [7] used an Envirosafe Alkali Activation Binder (AAB) instead of a traditional cement-based binder to stabilize black cotton soil (BCS). Shashank Gupta et al. [8] have developed a fly ash-based process that is economical, ecologically safe, and significantly improves the engineering and strength properties of jute as a geotextile. Noor Aamer Odeh et al. [9] evaluate the mechanical, durability, and microstructure properties of clayey soil (with different percentages of sand) stabilized using a coal-fired fly and a sodium hydroxide/sodium silicate solution alkali activator. At present, research on geopolymers is mainly focused on cementing materials and concrete, but there is little research on the effect of geopolymers on the strength of improved soil. A geopolymer binder is a kind of green cementitious material with fast hardening strength, a high shrinkage rate and low acid and alkali corrosion resistance. Sart Sukprasert et al. [10] found that the UCS values of a fly ash-based geopolymer stabilized by Silty Clay and Blast Furnace slag blend increased with the increase in NaOH concentration. Additionally, high concentrations of NaOH can dissolve silica and alumina, which react with NaOH to produce geological polymerization. In strong alkaline solutions, the aluminosilicates are rapidly dissolved to form the free SiO_4_ and AlO_4_ tetrahedra. The SiO_4_ and AlO_4_ tetrahedra will thereafter establish a range of alternative spatial arrangements that can be grouped into three types of monolithic geopolymer products [11]. Rinu Samuel et al. [12] found that a geopolymer has a significantly lower carbon footprint than lime or cement binders.

Wang et al. [13] found that the contents of both the metakaolin and alkali-activator have significant impacts on the strength performance of the geopolymer-improved soil.

The synthesis of the geopolymer requires an aluminosilicate-rich source or precursor, an alkali-metal cation source (such as NaOH, KOH, or Ca(OH)_2_), an additional source of silica (as needed), and water [14]. In a study by Bikash Adhikari et al., the UCS of the soil-geopolymer decreased with the addition of sodium silicate, and the results were similar for high and medium plastic soils in the study, suggesting that sodium hydroxide alone could be used in soil-geopolymer mixtures to improve strength properties [15]. Bikash Adhikari et al. concluded that sodium silicate had no effect on the strength development of soil-geopolymer mixtures [15]. V. Gokul et al. reported [16] the effect of different alkali-to-binder ratios on the strength of treated soil.

Before the test, the XRD test showed that the original soil used in this test provided SiO_2_, so sodium silicate or silica fume will not be added in this study. Sodium hydroxide alkali activates metakaolin to solidify soil, which is used as a modifier of subgrade materials to improve soil.

## 2. Materials and Methods

### 2.1. Experimental Materials

#### 2.1.1. Soil

This study used clay from Anhui place into an oven to dry at a low temperature to prevent high temperature damage from the clay mineral composition; the oven temperature was set to 105 °C, with a drying time of 12 h. Before sampling, they were crushed and placed in a 2 mm sieve to discard the remaining parts of the clay. The physical properties of the original soil were determined according to GB/T50123-2019 (Chinese) [17]. The Index properties of clay soil is shown in Table 1.

#### 2.1.2. Metakaolin

The main oxides of metakaolin used in the experiment are SiO_2_ and Al_2_O_3_, and their total content is about 98%. Its 28-day activity index is 123% and loss on ignition (LOI) is 13.7%. D50 is 3.36 µm. Metakaolin was obtained by calcination of typical clay minerals (kaolin) in the temperature range of 650–800 °C Metakaolin has a complex amorphous structure and is the product of de-hydroxylation of 1:1 clay mineral kaolinite. The chemical reaction of kaolinite forming Metakaolin is as follows [18]:

2 Al_2_Si_2_O_5_(OH)_4_  →  Al_2_Si_2_O_7_ + 2 H_2_O 
Kaolinite                    Metakaolin

Compared with other materials, MK is considered to be environmentally friendly and cost-effective. The chemical composition of metakaolin by EDX analysis is shown in Table 2.

#### 2.1.3. Alkali-Activator

Generally, NaOH or KOH and sodium silicate are combined to stimulate the activity of the precursor. Therefore, NaOH is selected because due to the high price of KOH. In order to explore whether SiO_2_ can be provided in clay to participate in the polymerization of geopolymers, this paper did not add alkali activators such as sodium silicate that can provide SiO_2_. Hydroxide sodium is an organic compound with the chemical formula NaOH. The sodium hydroxide used in this article is analytical grade, which is in flake form. The sodium hydroxide was dissolved in distilled water by a specified weight to achieve the desired molar concentration weight. Before adding the mixture (NaOH + H_2_O) to metakaolin to prepare the geopolymer, the mixture (NaOH + H_2_O) was allowed to sit to allow the heat to dissipate.

### 2.2. Sample Preparation

Each material ratio is a group; at least three samples were prepared per group to ensure the higher reliability of the experimental data. MK was added in proportion to 4%, 8%, 10% and 12% of the total dry weight of the soil and metakaolin. The alkali activator-to-ash ratio was maintained at 0.7. When the ratio of alkali activator-to-ash is 0.6 or less, there is still a lot of ash not soaked in the alkali activator. When the ratio is 0.8 or more, the geopolymer at this time is too watery as a binder, which is not conducive to bonding soil. Therefore, 0.7 was selected as the fixed alkali-cement ratio, and this variable was fixed. The optimum moisture content of soil was determined to be 12.76% by the compaction test. The required amount of water to be added to the dry soil to achieve its optimum water content (OMC) was added to the MKG mixture but not to the dry soil, then the diluted MKG mixture was added to the dry soil and mixed thoroughly for about 5 min. Then, the soil was sealed in a container for at least 12 h to equilibrate. Meanwhile, a selection of plain soil was prepared as the control group. Because the use of geopolymers for soil improvement has only been studied in recent years, there are no standards or technical specifications. The experiment scheme is shown in Table 3.

**Figure 1:**
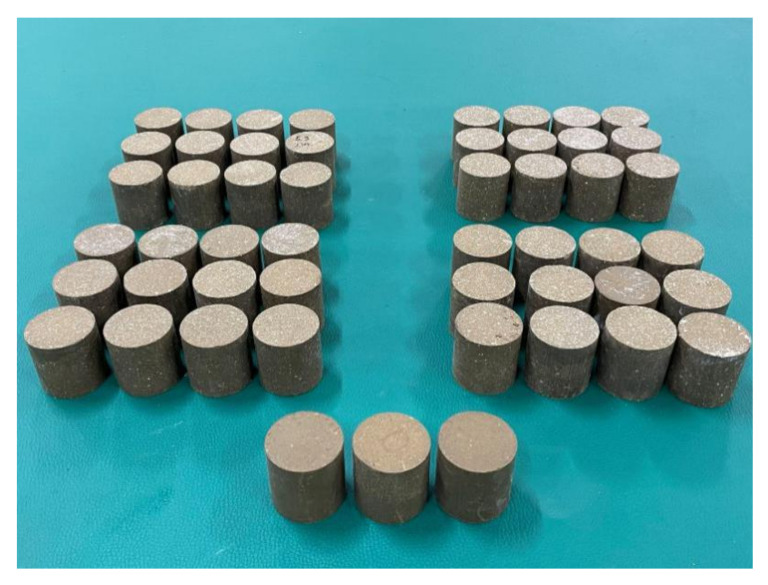
A set of prepared samples.

The fully mixed soil was put into a cylindrical metal mold with a diameter of 50 mm and a height of 50 mm. The surface was coated with Vaseline. Then, the test mold was placed on the jack in the reaction frame and pressurized at a loading rate of 1 mm/min until the upper and lower pressure columns are pressed into the test mold. The pressure was maintained for 2 min. Then, the prepared sample was put into a sealed bag in constant temperature and humidity (temperature: 20 °C, relative humidity: 95%) curing for 3, 7, 14 and 28 days. Figure 1 is a set of prepared samples.

## 3. Result and Discussion

### 3.1. Unconfined Compression Strength

UCS is an important parameter used to determine the minimum standard to be used as pavement material to support construction and normal traffic. According to the Standard Test Method for the Unconfined Compressive Strength of Clay [19], an axial load was applied to the sample until failure at a rate of 1% mm/min. The stress and strain of the specimen were recorded at intervals of 5 s until the specimen failed.

#### 3.1.1. UCS of Soil-Geopolymer

**Figure 2:**
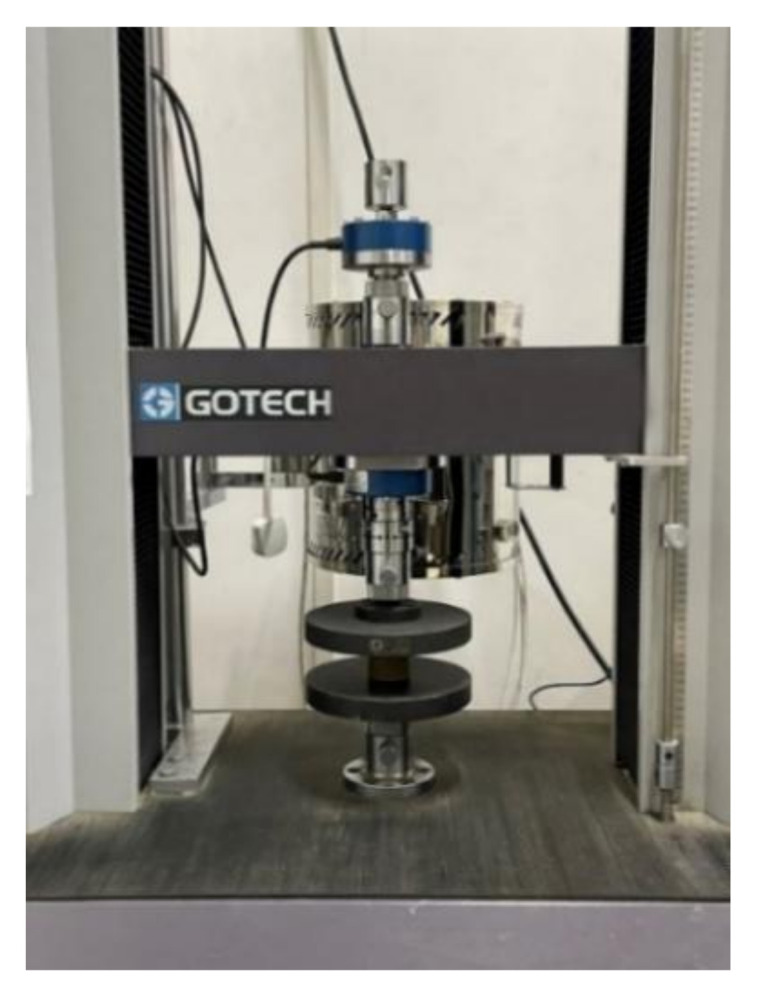
Unconfined compressive strength test.

Figure 2 is the instrument for testing UCS. The stress–strain curve was obtained through the test in order to determine the compression load value when the sample is broken. UCS was computed using the equation as shown:

$$\mathrm{USC}=\frac{P}{A}$$

where P: maximum load to failure (N) and A: cross sectional area of specimens (mm^2^).

The samples were tested 3, 7, 14, and 28 days after curing.

**Figure 3:**
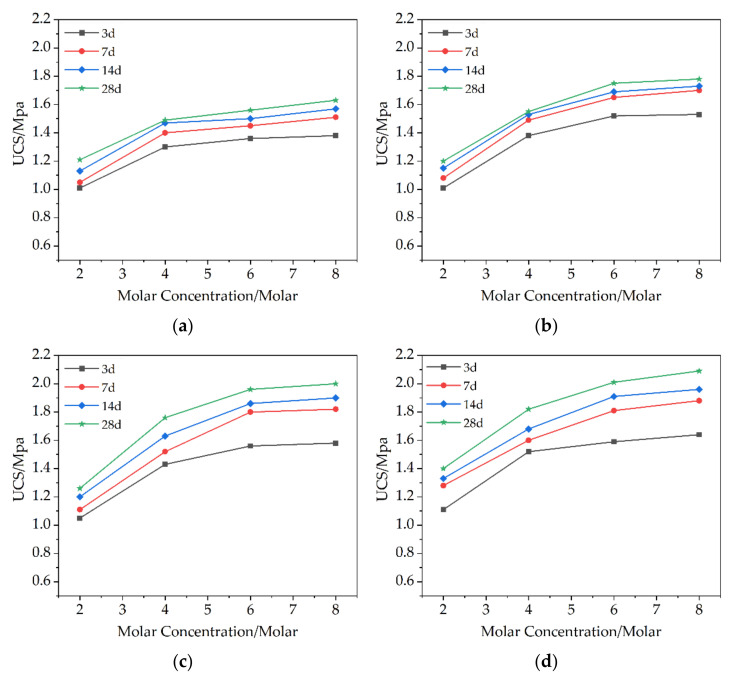
UCS values of different MKG contents: (**a**) result for 6% content of MKG; (**b**) result for 8% content of MKG; (**c**) result for 10% content of MKG; (**d**) result for 12% content of MKG.

The unconfined compressive value of the untreated soil is about 1938 kPa. Figure 3 shows the unconfined compressive strength of different MKG contents. The results showed that the samples with high molar concentration showed higher strength and the strength increased with the increase in time, reaching the maximum value at 28 days of curing. In addition, the strength of the clay also increased with the increase in geopolymer content. In this study, the strength of the M12A8 group was the highest. The unconfined compressive strength of the M12A8 group can reach 2.09 Mpa. Mo Zhang et al. [2] concluded that the compressive strength of MKG stabilized soils increased with the concentration of MKG. Shams O. Abdulkareem et al. [18] found that the unconfined compressive strength of soft soil increases with the addition of MK.

Due to the better alkaline environment provided by the higher molar concentration of the alkali activator, with the increase in the amount of alkali activator, the active substance will be fully dissolved, and the active substance SiO_2_ and Al_2_O_3_ in the soil will be more completely polymerized. The results show that when the molar concentration is 2M, the strength does not increase obviously, when the molar concentration is increased to 4M, the strength increases significantly. When the molar concentration of NaOH reaches 4M, the strength increases with the molar concentration of NaOH, but the strength does not increase significantly. Other studies have had similar results. Bikash Adhikari et al. [15] concluded that the strength of the soil-geopolymer mixture increased with increased concentration of NaOH for the medium plastic soils. However, high plastic soils did not show much improvement with increased concentration of NaOH. Pooria Ghadir et al. [20] found that an increase in the molar concentration of sodium hydroxide from 4 to 12 M results in higher solubility of the silicoaluminate precursor, resulting in a higher yield of adhesive gel.

It shows that when the molar concentration of sodium hydroxide is increased to 4M, the active substance can be fully dissolved. When the molar concentration of sodium hydroxide is higher, the effect is not obvious and not economical.

As time goes on, the intensity increases. This result may be attributed to the gradual crystallization of new minerals structure in the reaction products. The results show that the unconfined growth rate of the treated samples is the fastest in the first seven days, and the growth rate gradually flattens out in the later days, which may be attributed to the rapid condensation of the geopolymer. With the increase in MKG content, the strength also increases.

#### 3.1.2. Failure Mechanism of Geopolymer-Improved Soil

**Figure 4:**
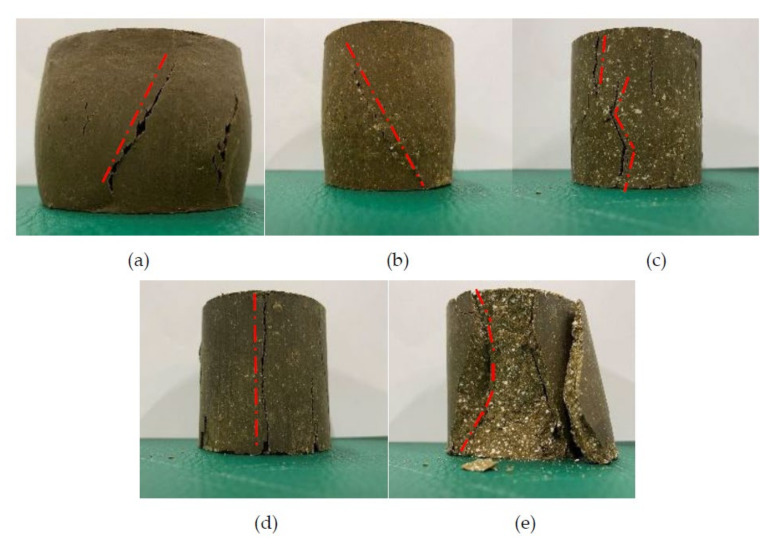
Failure modes of different groups of geopolymer-improved soil: (**a**) untreated soil; (**b**) M6A8; (**c**) M8A8; (**d**) M10A8; (**e**) M12A8.

It can be seen from Figure 4 that the soil treated in different ways has different destruction forms. Failure patterns of untreated soil and soil samples with different geopolymer contents are shown. Figure 4a shows an image of the failure mode of untreated soil. It is plastic failure, showing a waist drum shape. Figure 4b shows the failure mode image of group M6A8. This group of samples had the same plastic failure as the untreated soil, with obvious shear bands. In Figure 4c, the failure mode of group M8A8 is vertical expansion cracking. Figure 4d shows that the failure mode of group M10A8 is a vertical split failure. In Figure 4e, the failure mode of group M12A8 was tapered in the middle when destroyed.

The improved soil with low MKG content usually has obvious shear zone. In this case, the failure mode of the improved soil with MKG is essentially the plastic failure of the soil in the shear zone. Soil treated by high MKG content is brittle failure, which may change to brittle failure because the brittleness of the geopolymer itself will lead to a decrease in soil plasticity.

In the study of Wang et al. [13], similar results were obtained as in this paper. The geopolymer-improved soil with low metakaolin and alkali-activator contents commonly has an obvious shear band. As the content of the metakaolin and alkali activator increased, the formation of shear bands began to be less obvious than before. The damage of geopolymer-modified soil gradually changed to vertical bulging cracking and splitting cracking. When the alkaline environment provided by the alkali activator is poor, the polymerization of active substances SiO_2_ and Al_2_O_3_ in soil is not complete. With the increase in the amount of alkali activator, the active substances will be fully dissolved and polymerized. Therefore, the brittleness of the geopolymer itself will lead to a reduction in the overall toughness of the soil.

### 3.2. Test of Water Resistance

**Figure 5:**
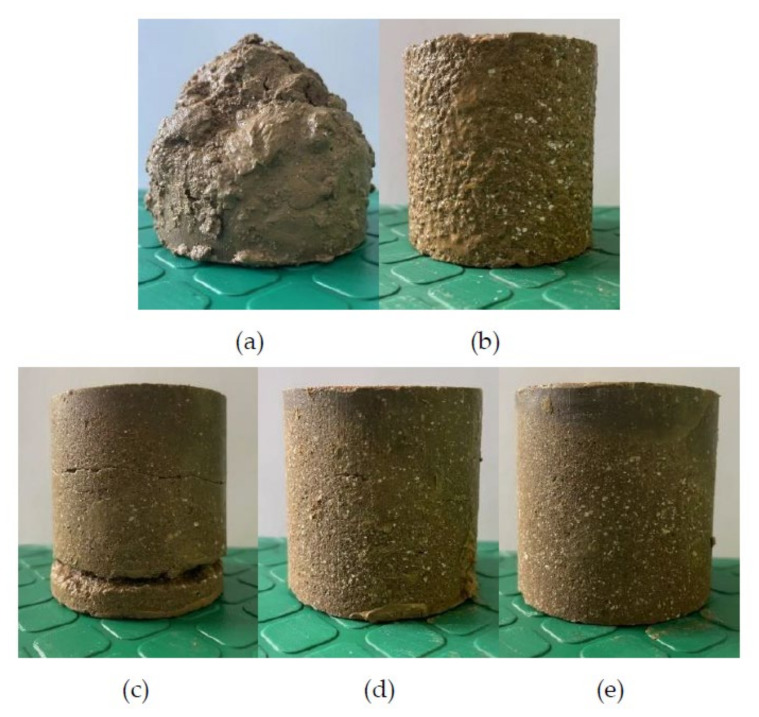
Sample soaked in water: (**a**) untreated soil; (**b**) M6A8; (**c**) M8A8; (**d**) M10A8; (**e**) M12A8.

After curing for 28 days, the samples were taken out of the curing box and soaked in water at 20 °C for 24 h. The surface of the sample was also 2 cm from the water. From Figure 5a–e, the untreated soil, M6A8, M8A8, M10A8 and M12A8 are shown in the in order. In Figure 5a, the untreated soil is severely damaged after soaking. It can be seen from the figure that with the increase in MKG content, the integrity of samples soaked in water is better. Figure 5e shows that the group M12A8 performed best, remaining intact after immersion.

In the study of Yu J et al. [21], by increasing the curing time, the curing agent reacts with soil moisture to produce more hydration products, thus improving the density of the samples and water resistance. At the same time, this good water resistance prevents the solidified soil from being soaked by rain.

### 3.3. Microstructure Characteristics of Geopolymer-Improved Soil Using SEM and XRD

As the M12A8 group of samples performed best in the unconfined compressive strength tests, scanning electron microscopy (SEM) and X-ray diffraction (XRD) tests were performed on this group of samples and pure soil. In order to qualitatively observe changes in soil particles, colloidal morphology and other microstructures, the microstructure of pure clay and M12A8 cured for 28 days was observed by SEM.

**Figure 6:**
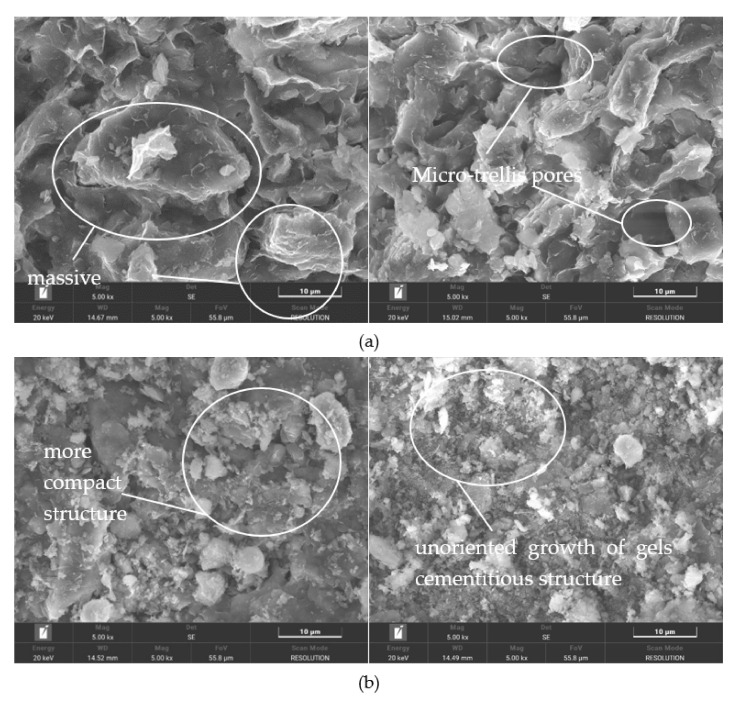
SEM images: (**a**) pure soil; (**b**) treated soil.

Figure 6 shows micrographs of untreated and treated soil magnified 5000 times. In Figure 6a, scanning electron microscopy showed that loose massive, disordered elements dominated the untreated clay. Weak strength is due to a large number of voids in untreated soil. Figure 6b shows the treated soil; geopolymers were found to react with the clay, covering the clay particles with a binder. The silicoaluminate gel formed by the soil-geopolymer enhanced the connection between soil particles and, thus, enhanced the mechanical properties of the clay. So, the treated soil had a denser, more compact structure than the untreated soil.

Shams O. Abdulkareem et al. [18] concluded that the microstructure of the mixture without Metakaolin-based Geopolymer additive is loose and contains a lot of void pores. Geopolymer gels were formed in the treated soil. Wang et al. [13] found that the loose flaky units of the pure clay were wrapped and connected by unoriented gels with the development of curing time. The arrangement between the supper aggregates and the cementitious flocs were denser with a more homogeneous microstructure. In the study of Mo Zhang et al. [2], as the dosage of MKG increased from 0% to 15%, the uniformity of the microstructure is significantly improved, which explains why UCS increased with the increase in MKG concentration.

**Figure 7:**
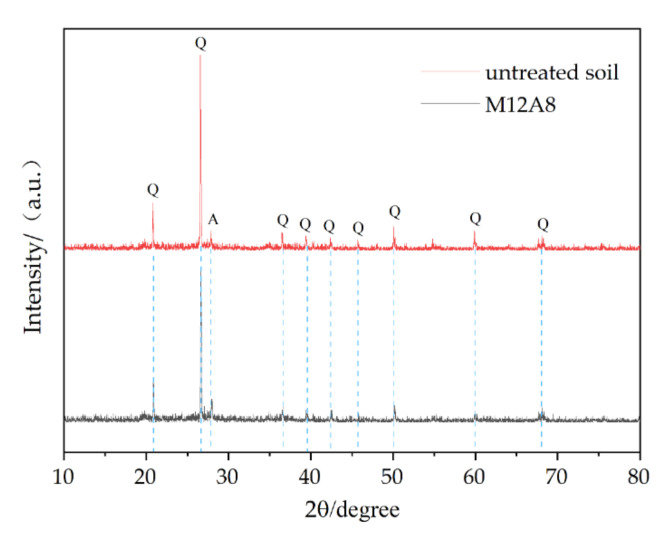
XRD patterns of Untreated soil and M12A8. Mineral abbreviations: Q: quartz; A: albite.

XRD analysis was performed on untreated soil and group M12A8 after curing for 28 days. The original soil and treated soil samples were dried and pulverized into powder in a mortar. The samples were sieved through 200 mesh and tested by XRD. It was performed over a 2θ range of 10°–80° at a 2.4°/min scanning rate. The purpose of the XRD test was to determine whether the geopolymer reacts with the soil by observing the formation of any peaks. Figure 7 is the XRD patterns of Untreated soil and M12A8. The XRD reflection intensity of quartz was lower than that of untreated soil. The reason is that at higher pH values, the presence of such ions as Na+ accelerates the dissolution of quartz and amorphous silica. The XRD reflectance of the remaining minerals showed almost the same strength as the untreated soil, indicating that there was no direct chemical reaction between the geopolymer precursor and them. The reduction in quartz content indicated that the clay provides a source of silica for the formation of a geopolymer.

M12A8 has better mechanical properties than all the other samples due to its higher content of geopolymers, its ability to fill particles between clay particles, and its denser structure. For example, from M12A2 to M12A8, its mechanical properties gradually increased. The reason for this is that the silica provided by metakaolin and clay is sufficiently dissolved in strong alkali conditions, and M12A8 produces more cementing components than other samples and binds soil particles together to increase strength. The reaction of MKG with clay resulted in the formation of adhesive particles, which can be observed in the treated soil. Because more geopolymers fill the pores in the clay, M12A8 has better mechanical properties than other samples with less geopolymers, such as M6A8.

In a study by Mahmoud A. Mahrous et al. [11], the results also showed that the XRD reflection intensity of quartz decreased under OMC and saturated conditions with MKG and FAG compared with natural soil. His explanation is that the presence of cations Na+ accelerates the dissolution of quartz and amorphous silica due to the release of higher concentrations of Na+ from the geopolymer at higher pH values. In the study of Gokul V et al. [16], it is beneficial to add NaOH and GGBS to clay. The unconfined compressive strength (UCS) of stabilized soils increases with the increase in GGBS percentage, which is similar to the results in this study. Shams O. Abdulkareem et al. [18] concluded that the unconfined compressive strength of soft soil increases with the addition of MK. By increasing the curing time from 1 day to 14 days, the unconfined compressive strength can be significantly improved. In the study of Mo Zhang et al. [2], the UCS value of MKG stabilized soil was much higher than that of soil, and when MKG concentration was higher than 11%, it was higher than that of 5% PC stabilized soil. However, the increase in strength from 7 days to 28 days of curing was not significant, which is similar to the results in this paper.

## 4. Conclusions

(1) The unconfined compressive strength of clay increases with the increase in the metakaolin-based geopolymer and the value is 4109 kN when MKG content is 12%.
(2) As the curing time increases from 3 days to 28 days, the unconfined compressive strength continues to increase, but tends to moderate. The increase was faster in the first 14 days. This may be due to the rapid reaction of MKG precursor.
(3) The molar concentration of sodium hydroxide is too low to fully dissolve the active substance. When the molar concentration reaches 4M, the strength is significantly improved, while the higher concentration of sodium hydroxide does not greatly improve the strength.
(4) With the increase in MKG and the molar concentration of the alkali activator, the failure of treated soil changed from plastic failure to brittle failure.
(5) Scanning electron microscopy demonstrated that the treated soil has a denser structure, and the clay particles are covered with gelling compounds produced by the geopolymer, which contributes to its strength.
(6) X-ray diffraction showed that clay provides a source of silica for the formation of geopolymers.

## Figures and Tables

**Table 1:** Index properties of clay soil.

Property	Specific Gravity	Liquid Limit (%)	Plastic Limit (%)	Plasticity Index (%)	Maximum Dry Density (g/cm^3^)	Optimum Moisture Content (%)
Index value	2.65	41.52	13.85	27.67	1.91	12.81

**Table 2:** Chemical compositions of raw materials metakaolin.

Chemical Composition	SiO_2_	Al_2_O_3_	Fe_2_O_3_	TiO_2_	CaO	MgO	K_2_O	Na_2_O
Ratio (%)	54.31	43.73	0.53	0.67	0.26	0.19	0.08	0.22

**Table 3:** Experiment scheme.

Metakaolin Content	Alkali-Activator (Sodium Hydroxide with Different Molar Concentrations)			
	2	4	6	8
6	M6A2	M6A4	M6A6	M6A8
8	M8A2	M8A4	M8A6	M8A8
10	M10A2	M10A4	M10A6	M10A8
12	M12A2	M12A4	M12A6	M12A8

## Data Availability

Not applicable.

## Sample Availability

Soil samples are not available from the authors.
